# Statistical methods for discrimination of STR genotypes using high resolution melt curve data

**DOI:** 10.1007/s00414-024-03289-x

**Published:** 2024-07-13

**Authors:** Darianne C. Cloudy, Edward L. Boone, Kristi Kuehnert, Chastyn Smith, Jordan O. Cox, Sarah J. Seashols-Williams, Tracey Dawson Green

**Affiliations:** 1 Department of Forensic Science, Virginia Commonwealth University, 1015 Floyd Avenue, PO Box 843079, Richmond, VA 23284 USA; 2 Department of Statistical Sciences and Operations Research, Virginia Commonwealth University, 1015 Floyd Avenue, PO Box 843079, Richmond, VA 23284 USA

**Keywords:** High resolution melt curves, qPCR genotyping, Prediction modeling, Principal component analysis (PCA), Linear Discriminant Analysis (LDA)

## Abstract

Despite the improvements in forensic DNA quantification methods that allow for the early detection of low template/challenged DNA samples, complicating stochastic effects are not revealed until the final stage of the DNA analysis workflow. An assay that would provide genotyping information at the earlier stage of quantification would allow examiners to make critical adjustments prior to STR amplification allowing for potentially exclusionary information to be immediately reported. Specifically, qPCR instruments often have dissociation curve and/or high-resolution melt curve (HRM) capabilities; this, coupled with statistical prediction analysis, could provide additional information regarding STR genotypes present. Thus, this study aimed to evaluate Qiagen’s principal component analysis (PCA)-based ScreenClust^®^ HRM^®^ software and a linear discriminant analysis (LDA)-based technique for their abilities to accurately predict genotypes and similar groups of genotypes from HRM data. Melt curves from single source samples were generated from STR D5S818 and D18S51 amplicons using a Rotor-Gene^®^ Q qPCR instrument and EvaGreen^®^ intercalating dye. When used to predict D5S818 genotypes for unknown samples, LDA analysis outperformed the PCA-based method whether predictions were for individual genotypes (58.92% accuracy) or for geno-groups (81.00% accuracy). However, when a locus with increased heterogeneity was tested (D18S51), PCA-based prediction accuracy rates improved to rates similar to those obtained using LDA (45.10% and 63.46%, respectively). This study provides foundational data documenting the performance of prediction modeling for STR genotyping based on qPCR-HRM data. In order to expand the forensic applicability of this HRM assay, the method could be tested with a more commonly utilized qPCR platform.

## Introduction

Over the past two decades, methods for forensic DNA analysis have greatly increased in efficiency, sensitivity, and accuracy. However, samples with a limited number of DNA copies continue to create challenges for forensic laboratories. The stochastic effects associated with low template (Lt) DNA, such as STR allele drop-out, allele drop-in, and inter-locus/intra-locus peak imbalances, can cause uncertainty in the interpretation process [1]. In addition to the difficulty of interpreting Lt DNA profiles, the presence of DNA from multiple contributors can further complicate the data, increasing uncertainty [2]. Adjustments to procedures that could compensate for these issues and potentially yield more discernable profiles are often not possible, as artifacts and other profile characteristics are not revealed until the end of the DNA workflow. In order to correct these issues, the analyst may need to return to the sample preparation, DNA extraction, or STR amplification stage, which is costly, time-consuming, and may not be possible if the sample was consumed in the initial testing. Thus, having more information earlier on in the analytical workflow would make better use of analyst time and maximize the utility of limited evidentiary samples.

Melt curve analysis utilizing qPCR instrumentation has long been explored as a potentially useful tool for a variety of forensic applications including mRNA analysis for body fluid identification, species identification, individualization of twins via methylation pattern analysis, Y STR screening, and human identification via single nucleotide polymorphism (SNP) analysis [3–10]. Recently, Torres et al. (2023) developed a high-resolution melt (HRM) assay that, when combined with the Quantifiler™ Trio kit (Thermo Fisher Scientific, Waltham, MA) components and machine learning algorithms, was capable of accurately predicting if a forensic sample is a single-source or mixed DNA sample for 79% of samples tested [10]. However, the assay was unable to predict the number of contributors (for mixture samples) nor was it able to determine the contributor genotypes [10, 11]. Introducing a detection assay to the quantification step that could identify contributor genotype and determine the number of contributors present would significantly strengthen this application. For example, having access to minimal STR genotyping information at the DNA quantification step would allow the examiner time to adjust the workflow prior to STR multiplex amplification. Further, this would allow them to provide early exclusionary information to investigators, if genotypes can be properly resolved.

Melt curve analysis of STR amplicons has been explored for *rapid genotyping* of single source DNA samples [12–18]. For example, one UK study explored amplification of the D18S51, TH01 and D8S1179 loci using HyBecon^®^ fluorescent probes and non-fluorescent blocker oligonucleotides to enhance melt curve analysis; this study successfully obtained partial STR profiles from buccal swabs [13]. Additionally, Kuehnert et al. developed an optimized procedure for the amplification of D5S818 and D18S51 with subsequent high-resolution melting (HRM) using a Rotor-Gene^®^ Q (QIAGEN, Hilden, Germany) and an intercalating dye (EvaGreen^®^) [12]. While distinguishable peaks for each STR locus were observed from resulting melt curve data, genotypes were not consistently discernible [12]. Further, this assessment only analyzed 16–20 samples, all having one of only three closely related STR genotypes. Similarly, Nguyen et al. [14] developed a melt curve screening tool for forensically relevant samples utilizing mini-STR primers for CSF1PO and TH01 along with either Taqman^®^ chemistry or intercalating dyes (SYBR^®^ Green or LCGreen^®^ Plus). The study reported accurate STR allele determination from degraded and inhibited samples, but noted inconsistent reproducibility of the assay [14]. Unfortunately, none of these studies reported exploration of statistical software-based models, which could help improve accuracy and remove subjectivity from melt curve analysis.

The Qiagen Rotor-Gene^®^ ScreenClust HRM^®^ software (Qiagen) incorporates principle component analysis (PCA) as a way to group like-samples using melt curve data [12, 19, 20]. PCA is a correlational technique which transforms data into its main elements; from this transformation and reduction in dimension, a linear combination of variables can create new data. The newly created data can then be assessed for underlying patterns and variation [21]. Alternatively, linear discriminant analysis (LDA) is a classification algorithm that attempts to make a distinction *between* observations. LDA assesses the data provided and compares it to other previously classified data patterns by determining similarity based on variance between classes and within classes [12, 22, 23]. As of today, there is not a packaged software available for high resolution melt curve analysis which utilizes an LDA-type classification algorithm; however, previous work has generated code in R statistical software (©The R Foundation, Vienna, Austria) to meet this goal [12, 24]. Further exploration of HRM analysis for STR genotyping should include an analysis of a wide range of STR genotypes as well as a quantitative assessment of the melt curve data using statistical prediction modeling in order to determine if HRM could be used to provide reliable STR genotyping information for forensic investigations.

## Methods and materials

### Sample collection & initial DNA analysis

This study utilized previously collected DNA samples as well as buccal swab samples collected from volunteers in compliance with Virginia Commonwealth University Institutional Research Board protocol number HM20002931 and HM20006066. DNA from newly obtained samples was extracted using a QIAcube liquid handling robot (QIAGEN, Hilden, Germany) and the standard manufacturer’s Buccal Swab Spin QIAcube Protocol using QIAamp^®^ DNA Blood Mini kit reagents (Qiagen). Extracted samples were quantified using manufacturer’s protocol, but with half-volume reactions using the Investigator^®^ Quantiplex Kit (Qiagen) on the Rotor-Gene^®^ Q (Qiagen). Reference STR profiles for each sample were developed by amplifying 1ng of DNA extract with the AmpFLSTR^®^ Identifiler^®^ PCR amplification kit (Thermo Fisher Scientific, Waltham, MA) on the GeneAmp 9600 thermal cycler (PerkinElmer, Waltham, MA). The 15 µl reaction consisted of 5.7 µl of PCR Reaction mix, 2 µl of Primer set, 2.1 µl Tris-EDTA (TE), 0.2 µl of AmpliTaq Gold™ Polymerase (5U/µl) (Applied Biosystems, Waltham, MA), and 5 µl of template DNA. Thermal cycling conditions included a pre-denaturing step at 94 °C for 11 min, followed by 28 cycles of: denature 94 °C for 1 min, anneal 59 °C for 1 min, extension 72 °C for 1 min, and final post-extension step of 60 °C for 90 min. Amplified STR products were then separated and detected on the ABI PRISM^®^ 3130 Genetic Analyzer (Thermo Fisher Scientific) using a 36 cm capillary array with a 10s injection. Each reaction consisted of 0.1 µl of GeneScan™ 500-LIZ™ size standard (Thermo Fisher Scientific) and 12 µl of Hi-Di™ formamide (Thermo Fisher Scientific) diluent. The wells containing an allelic ladder received 1 µl of the ladder; 1.5 µl of amplified DNA was added to all other sample wells. The profiles were analyzed using GeneMapper ID™ software v4.1 (Thermo Fisher Scientific) with an analytical threshold of 75 relative fluorescent units (RFUs). The D5S818 and D18S51 genotypes were documented as the known reference genotypes for comparison in all studies detailed below. Ultimately, 311 samples were obtained and selected for this study. Samples selected were those that were available at the time of testing and had one of seven closely-related D5S818 genotypes [(10,11), (11,11), (11,12), (11,13), (12,12), (12,13), (13,13)] *and/or* one of six closely-related D18S51 genotypes [(12,14), (12,15), (12,16), (13,14), (13,16), (14,15)].

### STR locus amplification & melt curve detection

Samples selected were amplified for each of two STR loci (D5S818 and D18S51) separately on the Rotor-Gene^®^ Q using the primer and amplification parameters previously established [12]. Each amplification reaction included 1X concentration of AmpliTaq Gold™ Buffer, 3mM MgCl_2_, 250µM dNTPs, 1µM each of forward and reverse primer, 2U AmpliTaq Gold DNA polymerase, 1X concentration of EvaGreen^®^ intercalating dye (Biotium, Fremont, CA), and 250ng/µl of bovine serum albumin (BSA) in water. Two microliters of template DNA were added to each reaction for a total reaction volume of 40µl. Primer sequences for D5S818 were (F) 5’-GGGTGATTTTCCTCTTTGGT-3’ and (R) 5’-AACATTTGTATCTTTATCTGTATCCTTATTTAT-3’; primer sequences for D18S51 were (F) 5’-CAAACCCGACTACCAGCAAC-3’ and (R) 5’-GAGCCATGTTCATGCCACTG-3’. The amplification cycling for both primer sets consisted of an initial 10 min 95 °C denaturation followed by 45 cycles of: 95 °C denaturation for 5s, 56 °C annealing for 20s, and 65 °C elongation for 30s with fluorescence detection at the 65 °C elongation step in the standard green channel. A cycle of 72 °C for 2 min, 95 °C for 20s, 55 °C for 20s and 56 °C for 2 min followed to transition into the melt phase. The amplicons were melted by 0.1 °C incremental increases in temperature from 60 to 95 °C. Each incremental step was held for 2s with fluorescent detection throughout the melt using the high-resolution melt curve detection channel.

### Genotype prediction analysis from HRM data

For PCA analysis, melt curve data generated from each sample at both STR loci were separately analyzed using the Rotor-Gene^®^ ScreenClust HRM^®^ software. For the D5S818 sample set, 56 samples were assigned as the training samples or “standards” based on their known genotypes; similarly, for the D18S51 sample set, 52 samples were assigned as the training samples or “standards”, with 7–10 samples per genotype for both loci. For each locus analyzed, all experimental samples were included as unknowns submitted for prediction analysis; the software placed each unknown into a genotype category based on highest probabilities given acceptable variability from the group mean. From the predicted clusters, confusion matrices were generated and then used to assess the software’s prediction accuracy (given as an overall percentage). From this, the percentage of misclassification for each genotype was determined and trends were identified. Geno-groups were formed based on these patterns of misclassification at each locus tested as well as the similarity of the genotype (and thus, amplicons produced). To subsequently evaluate the prediction accuracy using the identified geno-groups, the standard (training) samples were re-assigned in the software (as belonging to a geno-group, rather than a specific genotype), unknown samples were reanalyzed, and the newly predicted clusters were assessed for accuracy, as indicated above. Several different geno-grouping options were explored in order to determine the best option for the highest PCA-based prediction accuracy.

**Fig. 1:**
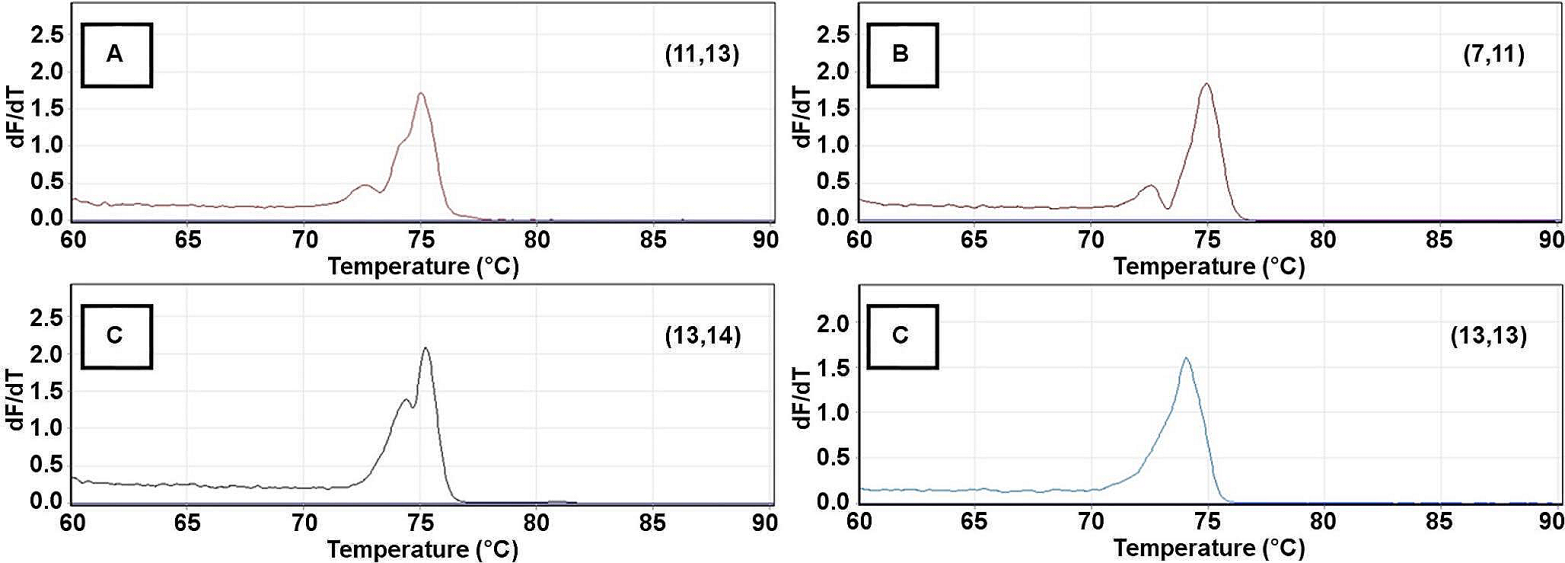
Representative D5S818 melt curves showing key morphological features used for LDA analysis. (**A**) Melt curve of genotype (11,13) with two shoulders present. (**B**) Melt curve of genotype (7,11) with one shoulder present that is fully resolved from the primary peak. (**C**) Melt curve of genotype (13,14) with one unresolved shoulder on the primary peak. (**D**) Melt curve of genotype (13,13) primary peak with no shoulders present

**Fig. 2:**
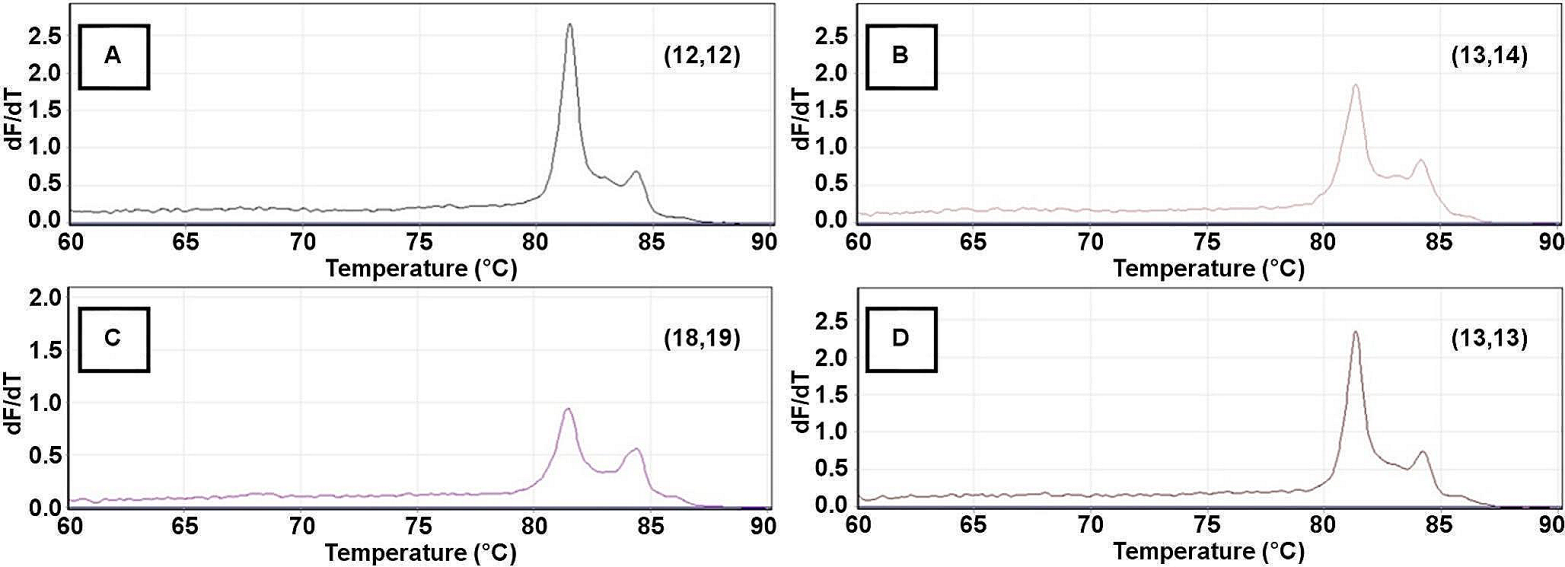
Representative D18S51 melt curves showing key morphological features used for LDA analysis. Melt curves from four samples (**A-D**) showing changes in the primary peak height: shoulder peak height ratios across different known genotypes

For LDA analysis, the melt curve data generated from each sample at both STR loci were separately analyzed using LDA code in R statistical software. The change in fluorescence (dF) with respect to temperature was exported, melt curves generated, and the primary peaks and shoulders were identified (Figs. 1 and 2). The data from each sample were then summarized into its primary peak and shoulder peak(s) temperatures along with their corresponding peak heights. For D5S818 samples, the peak/shoulder temperatures and peak heights for up to three observations were used; if only two peaks/shoulders were observed, the height at 64.95 °C was used as the third data point (required, as samples with disparate numbers of data points cannot be compared). No sample had fewer than two observed peaks. For D18S51 samples, the peak/shoulder temperatures and corresponding peak heights for four observations were used; if only three peaks/shoulders were observed, the peak height at 64.95 °C was used as the fourth data point. No sample had fewer than three observed peaks at this locus. For the D5S818 sample set, the same 56 samples used above were again assigned as the training samples for this analysis based on their known genotypes; similarly, for the D18S51 sample set, the same 52 samples used above were assigned as the training samples for this analysis. Code was generated in R statistical software so that the accuracy of LDA-based predictions could be calculated. Confusion matrices were generated and then used to assess the LDA prediction accuracy (given as an overall percentage). From this, the percentage of misclassification for each genotype was determined, trends were identified, and geno-grouping options were created. Geno-groups were formed, training samples were reassigned, and unknown samples reanalyzed, as described above. Several different geno-grouping options were explored in order to determine the best option for the highest LDA-based prediction accuracy.

## Results and discussion

### D5S818

When using the Rotor-Gene^®^ Q ScreenClust HRM^®^ software to predict D5S818 genotypes from HRM data using a PCA approach, samples were classified correctly only 23.77% of the time (Table 1). Overall, samples with known homozygous genotypes were more likely to classify accurately (39.58%) than samples whose known genotypes were heterozygous (20.18%). Most often, misclassified homozygous samples were predicted as having another homozygous genotype, whereas heterozygous sample misclassifications were more evenly split among homozygous and heterozygous genotypes. Conversely, when using LDA in R statistical software to predict D5S818 genotypes from the HRM data, samples were classified correctly at a rate of 58.92% (Table 2), which is substantially higher than the PCA-based model and the random chance rate of 14.29% (one in seven). As with the PCA method, samples with known homozygous genotypes were more likely to classify accurately (65.08%) than samples whose known genotypes were heterozygous (55.74%).

**Table 1:** Classification of D5S818 genotypes using HRM data and the PCA-based Rotor-Gene^®^ Q ScreenClust HRM^®^ software

	Principal Component Analysis Predicted Genotypes							
		(10,11)	(11,11)	(11,12)	(11,13)	(12,12)	(12,13)	(13,13)
Known Genotypes*	**(10**,**11)**	**5**	1	1	3	1	0	2
	**(11**,**11)**	4	**14**	3	1	7	1	2
	**(11**,**12)**	7	0	**10**	9	14	10	17
	**(11**,**13)**	3	9	3	**3**	2	6	8
	**(12**,**12)**	1	7	0	1	**7**	4	1
	**(12**,**13)**	1	1	7	1	5	**5**	7
	**(13**,**13)**	0	1	0	0	4	2	**5**
	Accuracy Rate: 23.77%							

*n = 12–67 per genotype

**Table 2:** Classification of D5S818 genotypes using HRM data and LDA analysis using R statistical software

	Linear Discriminant Analysis Predicted Genotypes							
		(10,11)	(11,11)	(11,12)	(11,13)	(12,12)	(12,13)	(13,13)
Known Genotypes*	**(10**,**11)**	**2**	1	1	5	0	2	0
	**(11**,**11)**	1	**34**	1	0	1	2	0
	**(11**,**12)**	1	0	**35**	3	10	0	0
	**(11**,**13)**	0	1	3	**26**	1	1	0
	**(12**,**12)**	0	2	4	1	**7**	1	0
	**(12**,**13)**	0	9	7	4	3	**5**	2
	**(13**,**13)**	0	5	0	2	1	1	**0**
	Accuracy Rate: 58.92%							

*n = 9–49 per genotype

In an attempt to increase prediction accuracies, 13 different geno-groupings were created (based on the above trends and misclassification rates) and tested. As expected, geno-grouping improved classification accuracies, regardless of which geno-grouping option was used or algorithm tested (data not shown). The three geno-grouping options that produced the highest prediction accuracies for each prediction model used in this study were assessed using the converse method to allow for direct comparison (Table 3). Geno-group option A provided the highest LDA-based and highest overall prediction accuracy (81.0%). Alternately, the highest prediction accuracy achieved with the PCA-based method was 46.6% (option F). Regardless of geno-grouping option tested, LDA-based prediction modeling provided higher geno-grouping prediction accuracies. This result may be due to the fact that LDA aims to maximize the separation amongst classes, in order to heighten class discrimination.

**Table 3:** Genotype prediction accuracy rates for top performing **D5S818** geno-groupings obtained using two prediction models (PCA and LDA)

		Genotypes Included	PCA Accuracy Rate	LDA Accuracy Rate
Option A	Group 1	(10,11), (11,13)	37.9%	**81.0%**
	Group 2	(11,12), (12,12)		
	Group 3	(11,11), (12,13), (13,13)		
Option B	Group 1	(11,11)	41.3%	76.2%
	Group 2	(11,12), (12,12), (12,13)		
	Group 3	(10,11), (11,13)		
	Group 4	(13,13)		
Option C	Group 1	(11,11), (13,13)	46.1%	74.6%
	Group 2	(11,12), (12,12), (12,13)		
	Group 3	(10,11), (11,13)		
Option F	Group 1	(10,11), (11,13)	**46.6%**	72.4%
	Group 2	(11,11), (12,12)		
	Group 3	(11,12), (12,13), (13,13)		
Option H	Group 1	(10,11), (11,11)	42.2%	70.8%
	Group 2	(11,12), (12,12)		
	Group 3	(11,13), (12,13), (13,13)		
Option J	Group 1	(10,11), (11,12)	45.6%	65.4%
	Group 2	(11,11), (12,12)		
	Group 3	(11,13), (12,13), (13,13)		

### D18S51

In order to determine if a more polymorphic STR locus would be better for genotype predictions using the two selected models, the testing was repeated using primers targeting the D18S51 locus. With more common genotypes known and higher levels of heterozygosity reported for this locus [15], one may expect the melt curve resolution, and thus genotyping predictions, to improve. When using the PCA-based Rotor-Gene^®^ Q ScreenClust HRM^®^ software to predict heterozygous D18S51 genotypes from HRM data, samples were classified correctly 40.38% of the time (Table 4). Of the 62 samples that misclassified, 40.32% had one allele predicted accurately with the second allele off by only one-repeat unit (one allele value). Additionally, the samples expected to produce heterozygous amplicons with the greatest difference in base pair length (those with 12,16 genotypes) were the most likely to be classified correctly (57.14%). This result is not surprising as the amplicons with the greatest difference in base pair length correspondingly have the greatest difference in melting rates thus producing visually distinct melt curves when compared amongst melt curves with amplicons that are close in base pair length and thus have similar melting rates. When LDA was used to predict D18S51 genotypes from the HRM data, samples were classified at a rate similar to the PCA-based method (45.10%, Table 5). However, unlike the PCA model for D18S51, the data obtained using the LDA approach showed no discernible trends when misclassifications were closely examined.

**Table 4:** Classification of D18S51 genotypes using HRM data and the PCA-based Rotor-Gene^®^ Q ScreenClust HRM^®^ software

	Principal Component Analysis Predicted Genotypes						
		(12,14)	(12,15)	(12,16)	(13,14)	(13,16)	(14,15)
Known Genotypes*	**(12**,**14)**	**7**	3	2	5	1	2
	**(12**,**15)**	6	**6**	4	2	2	0
	**(12**,**16)**	2	2	**8**	1	1	0
	**(13**,**14)**	4	1	1	**7**	6	1
	**(13**,**16)**	0	1	0	1	**7**	5
	**(14**,**15)**	1	1	0	1	6	**7**
	Accuracy Rate: 40.38%						

*n = 14–20 per genotype

**Table 5:** Classification of D18S51 genotypes using HRM data and LDA analysis using R statistical software

	Linear Discriminant Analysis Predicted Genotypes						
		(12,14)	(12,15)	(12,16)	(13,14)	(13,16)	(14,15)
Known Genotypes*	**(12**,**14)**	**9**	1	1	4	4	1
	**(12**,**15)**	7	**6**	2	1	3	1
	**(12**,**16)**	1	1	**4**	1	0	7
	**(13**,**14)**	0	1	1	**7**	0	11
	**(13**,**16)**	0	0	0	1	**6**	5
	**(14**,**15)**	0	0	0	1	1	**14**
	Accuracy Rate: 45.10%						

*n = 12–20 per genotype

As with the D5S818 locus, D18S51 geno-grouping options were created based on observed trends and classification rates; eight different geno-groupings were tested using both prediction models. As described above for D5S818, geno-grouping improved classification accuracies when the PCA algorithm was used; for LDA, however, only half of the geno-grouping options tested resulted in favorable increases in prediction accuracies (data not shown). The three geno-grouping options that produced the highest prediction accuracies for each prediction model used in this study were assessed using the converse method to allow for direct comparison (Table 6). Geno-group option E provided the highest PCA-based and highest overall prediction accuracy (65.4%). The highest prediction accuracy achieved with the LDA-based method was very similar (63.5%, option G). This study suggests that PCA-based methods may work better for predicting genotypes of loci that have increased allele diversity, such as that observed with D18S51.

**Table 6:** Genotype prediction accuracy rates for top performing **D18S51** geno-groupings obtained using two prediction models (PCA and LDA)

		Genotypes Included	PCA Accuracy Rate	LDA Accuracy Rate
Option A	Group 1	(12,14), (12,15)	53.9%	51.9%
	Group 2	(12,16), (13,16)		
	Group 3	(13,14), (14,15)		
Option C	Group 1	(12,14), (12,15)	62.5%	40.4%
	Group 2	(12,16), (13,14)		
	Group 3	(13,16), (14,15)		
Option E	Group 1	(12,14), (12,15), (13,14)	**65.4%**	51.9%
	Group 2	(12,16)		
	Group 3	(13,16), (14,15)		
Option F	Group 1	(12,14), (13,14)	63.5%	42.3%
	Group 2	(12,15), (12,16)		
	Group 3	(13,16), (14,15)		
Option G	Group 1	(12,14), (12,15)	49.0%	**63.5%**
	Group 2	(12,16)		
	Group 3	(13,14), (13,16), (14,15)		

## Conclusion

This study evaluated the use of PCA- and LDA-based prediction modeling tools for their ability to distinguish between genotypes of two STR loci using HRM data obtained from the Qiagen Rotor-Gene^®^ Q qPCR platform. When assessing the D5S818 locus, the LDA model substantially outperformed the PCA model for predicting genotypes. This trend held true when like-genotypes were grouped together for prediction analysis into geno-groups with prediction accuracies exceeding 80%. However, when assessing a more polymorphic STR locus (D18S51) with a more heterogeneous sample set, the differences in prediction accuracies between the models tested were far less pronounced suggesting that the LDA-based method may work better for predicting homozygous genotypes. Regardless of method or locus tested, placing samples with closely aligned genotypes into geno-groups for classification results in improved prediction modeling, but fewer classification options would limit the forensic utility of an HRM-based assay, as DNA from different contributors will be less likely to be individualized. Ultimately, the data from this study suggests that the best prediction model for STR genotyping may differ from locus-to-locus, depending on the nature and complexity of the STR locus tested. Further, the inclusion of additional heterozygous genotypes in the training sets used to train the software may improve overall prediction rates, regardless of the testing model employed.

Considering additional factors may be helpful when selecting a prediction model to use for genotyping using HRM data. For example, the PCA-based ScreenClust HRM^®^ software is commercially available, requires no coding, and is easy to use. However, the software is proprietary and the principal components it utilizes for analysis are unknown. Further, the ScreenClust HRM^®^ software requires that all known (training) standards be run on the instrument at the same time as tested unknown samples to provide the most accurate clustering. This would be impractical for wholescale use in forensic settings and becomes highly impractical when assessing loci with large repeat ranges and many common genotypes. Alternatively, R statistical software is free and training set data is stored for use of classification of unknown samples subsequently and independently tested. However, it requires some initial programming and forensic implementation would require the development of a more user-friendly interface.

In conclusion, this study provides foundational data documenting the performance of prediction modeling for STR genotyping based on HRM data. In order to expand the forensic applicability of the HRM assay described herein, it may be useful to test it using more commonly utilized qPCR platforms, such as Thermo Fisher’s QuantStudio™, and potentially incorporate it into the previously described mixture detection assay [10]. Further, exploring other prediction models that use similar classification schemes to those used in this study but are designed to classify larger data sets (e.g., comprehensive melt curve data), such as support vector machines (SVM), may prove useful [25–27].

## Data Availability

The datasets generated during and/or analyzed during the current study are available from the corresponding author on reasonable request.
